# Efficacy analysis of percutaneous balloon compression therapy for trigeminal neuralgia guided by three-dimensional digital subtraction angiography navigation

**DOI:** 10.3389/fneur.2025.1686574

**Published:** 2025-11-20

**Authors:** Zhennan Xu, Hui Li, Mingfa Liu, Ke Xu

**Affiliations:** Department of Neurosurgery, Shantou Central Hospital, Shantou, China

**Keywords:** trigeminal neuralgia, percutaneous balloon compression therapy, digital subtraction angiography, retrospective study, foramen ovale

## Abstract

**Introduction:**

The aim of this study was to evaluate the clinical efficacy of percutaneous microballoon compression guided by three-dimensional digital subtraction angiography (DSA) navigation in treating primary trigeminal neuralgia.

**Methods:**

We retrospectively analyzed 40 patients with primary trigeminal neuralgia who underwent percutaneous microballoon compression under three-dimensional digital subtraction angiography navigation between January 2019 and April 2025. Post-operative pain relief was assessed 1 d, 1 month, and 3 months post-surgery.

**Results:**

All 40 patients underwent three-dimensional digital subtraction angiography dual-volume imaging and 3D-Dyna-computed tomography cranial reconstruction at the pre-puncture, needle placement, balloon positioning, and balloon inflation stages. We adjusted the balloon morphology during inflation, considering cranial reconstruction and balloon positioning, for optimization. Post-operatively, all patients showed significant pain relief: 38 achieved immediate pain resolution, one resolved at 3 days, and one at 2 weeks. Overall, all 40 patients developed facial numbness, two developed perioral herpes, one developed diplopia, and one experienced transient masseter muscle weakness; all these resolved with treatment. Two patients showed recurrence during follow-up.

**Discussion:**

Three-dimensional digital subtraction angiography navigation-guided percutaneous microballoon compression is a safe and minimally invasive technique for the treatment of trigeminal neuralgia. Real-time 3D-Dyna-computed tomography reconstruction enables the visualization of the balloon-petrous apex relationship, enhancing procedural precision and therapeutic outcomes.

## 1 Introduction

Primary trigeminal neuralgia, characterized by unilateral paroxysmal lancinating pain within the distribution of the trigeminal nerve, has an annual incidence rate of 2.1–27 cases per 100,000 persons and significantly impairs quality of life (1). For medication-refractory cases, surgical interventions, including microvascular decompression (MVD), percutaneous trigeminal ganglion balloon compression (PBC), and radiofrequency thermocoagulation (RFT), are indicated (2). Since its initial description by Mullan and Lichtor in 1983 (3), PBC has been the preferred therapeutic option for pharmacotherapy-resistant or surgically ineligible patients because it is minimally invasive, has a short procedural duration, and results in high remission rates (4). However, conventional C-arm fluoroscopy-guided procedures are constrained by operator-dependent accuracy and subjective balloon morphology assessment (5). Recent advancements in digital subtraction angiography (DSA)-enabled three-dimensional (3D) navigation technology have enabled real-time image reconstruction, precise foramen ovale (FO) targeting, and dynamic monitoring of balloon positioning, thereby addressing critical limitations of traditional methodologies (6). We aimed to evaluate the clinical efficacy of DSA-3D-navigated PBC in treating refractory trigeminal neuralgia.

## 2 Methods

### 2.1 Patients

Overall, we retrospectively evaluated 40 consecutive patients (20 men, 20 women; mean age 65.2 ± 9.1 years) who underwent DSA 3D-navigated percutaneous trigeminal ganglion balloon compression for refractory primary trigeminal neuralgia at Shantou Central Hospital between January 2019 and April 2025. Unilateral involvement was right-sided in 23 patients and left-sided in 17 patients; affected trigeminal branches included the V1 in three cases, V2 in four cases, V3 in 15 cases, V1+V2 in one case, V2+V3 in nine cases, and V1+V2+V3 in eight cases. Pre-operative treatment revealed that 32 patients were intolerant to pharmacotherapy or it was ineffective; of these, one patient failed MVD, one failed RFT, one failed sequential RFT+MVD, one failed gamma knife therapy, and two had PBC recurrence (Table 1). This study has been approved by the Ethics Committee of Shantou Central Hospital (Ethics Approval Number: [2025] Research 045).

**Table 1:** Clinical data of 36 patients with trigeminal neuralgia.

**Characteristic**	**Value (*n*)**	**Proportion (%)**
**Sex**		
Male	20	50.0
Female	20	50.0
**Age (years)**		
<65	17	42.5
≥65	23	57.5
**Side affected**		
Left	17	42.5
Right	23	57.5
**TN pain distribution**		
V1 only	3	7.5
V2 only	4	10.0
V3 only	15	37.5
V1+V2	1	2.5
V2+V3	9	22.5
V1+V2+V3	8	20.0
**Prior surgical treatment**		
Treated with MVD	2	5.0
Treated with RFT	2	5.0
Treated with PBC	2	5.0
Treated with a gamma knife	1	2.5

MVD, microvascular decompression; PBC, percutaneous trigeminal ganglion balloon compression; RFT, radiofrequency thermocoagulation; TN, trigeminal neuralgia.

### 2.2 Surgical procedure

#### 2.2.1 Pre-operative preparation

All patients undergoing PBC fasted from food and fluids for 6 h pre-operatively. The patients were placed in a supine position under general intravenous anesthesia with orotracheal intubation. The endotracheal tube was secured to the contralateral side of the oral cavity relative to the surgical site and stabilized using an oral airway. Pre-operative cranial Dyna-CT (GE) 3D reconstruction was performed in a 20-s DCT Head 70 kV mode with a 220° rotation range to acquire intracranial 3D volumetric data, which were subsequently transferred to a dedicated 3D workstation for reconstruction processing.

#### 2.2.2 Surgical procedure

After achieving satisfactory anesthesia, all patients underwent initial 3D-DSA cranial Dyna-CT 3D reconstruction. The optimal FO angle was selected based on the reconstructed cranial images. The projection angle of the DSA machine was transmitted to the C-arm, which was positioned under fluoroscopic guidance to confirm the location of the FO. Adjustments were made to maximize the cross-sectional view of the FO, after which the C-arm was stabilized. A percutaneous puncture of the trigeminal ganglion was created using the Hartel anterior approach. Puncture advancement was halted upon loss of resistance and fluoroscopy confirmed the placement of the cannula tip within the upper medial quadrant of the FO. In cases of a difficult puncture, dynamic real-time fluoroscopy was performed at the FO site. Following successful puncture, we verified the cannula tip positioning at the outer orifice of the FO using a second 3D-DSA cranial Dyna-CT reconstruction FO. We adjusted the cannula depth considering the FO anatomy and the C-arm was repositioned in the lateral view, aligning the bilateral external auditory canals with the petrous ridge. The cannula stylet was replaced with a slightly longer, angled (15°) one. It was inserted through the cannula and directed downward toward the slightly posterior aspect of the petrous apex (typically not exceeding 1 cm beyond the cannula), facilitating channel dilation for balloon passage. After stylet placement, a third 3D-DSA cranial Dyna-CT reconstruction was performed using image clipping tools to delineate the relationship between the stylet tip and the petrous ridge. Correlative verification was performed using original bone-window images from multiple angles. The stylet was withdrawn and a Qingyuan balloon catheter was advanced via the cannula to the petrous ridge junction. A final 3D-DSA cranial Dyna-CT reconstruction confirmed the balloon depth, with the proximal marker positioned at the trigeminal impression of the ipsilateral bone window or slightly posteriorly. The distal marker was aligned with the exit zone of the trigeminal nerve root (crossing the petrous ridge). The balloon was slowly inflated with 0.2 ml of contrast medium and monitored for optimal pear-shaped morphology. Inflation was discontinued when significant pressure elevation occurred and leakages were prevented by adjusting the three-way stopcock. Follow-up 3D-DSA reconstruction was used to verify the balloon position relative to the petrous ridge, ensuring compression of the trigeminal nerve trunk. The balloon compression was maintained for 120–180 s. Vital signs, including heart rate and blood pressure, were monitored continuously. Gentle, controlled movements were prioritized during puncture and balloon inflation. The procedures were paused when hemodynamic instability occurred and resumed only after stabilization.

### 2.3 Assessment criteria

The visual analog scale (VAS) was used to evaluate the therapeutic outcomes of 3D-DSA dual-volume reconstruction-guided PBC. VAS scores were recorded at four time points: 1 day pre-operatively, 1 day post-operatively, and 1 and 3 months post-operatively.

VAS scoring criteria were:

0–1 point = cured, no pain
2–3 points = significant relief, occasional mild pain
4–7 points = partial relief, persistent moderate pain
8–10 points = ineffective, persistent severe pain.

### 2.4 Follow-up

Post-operative outpatient and telephone follow-ups were conducted for 3–12 months, with an average of 6 months.

### 2.5 Statistical analysis

SPSS (version 25.0) was used to statistically analyze the data. $\bar{\text{X}}\pm \text{s}$ represents normally distributed quantitative data. For repeated measures, ANOVA was used to compare the VAS scores of patients before and after PBC at various time points. Statistical significance was set at *P* < 0.010.

## 3 Results

**Figure 1:**
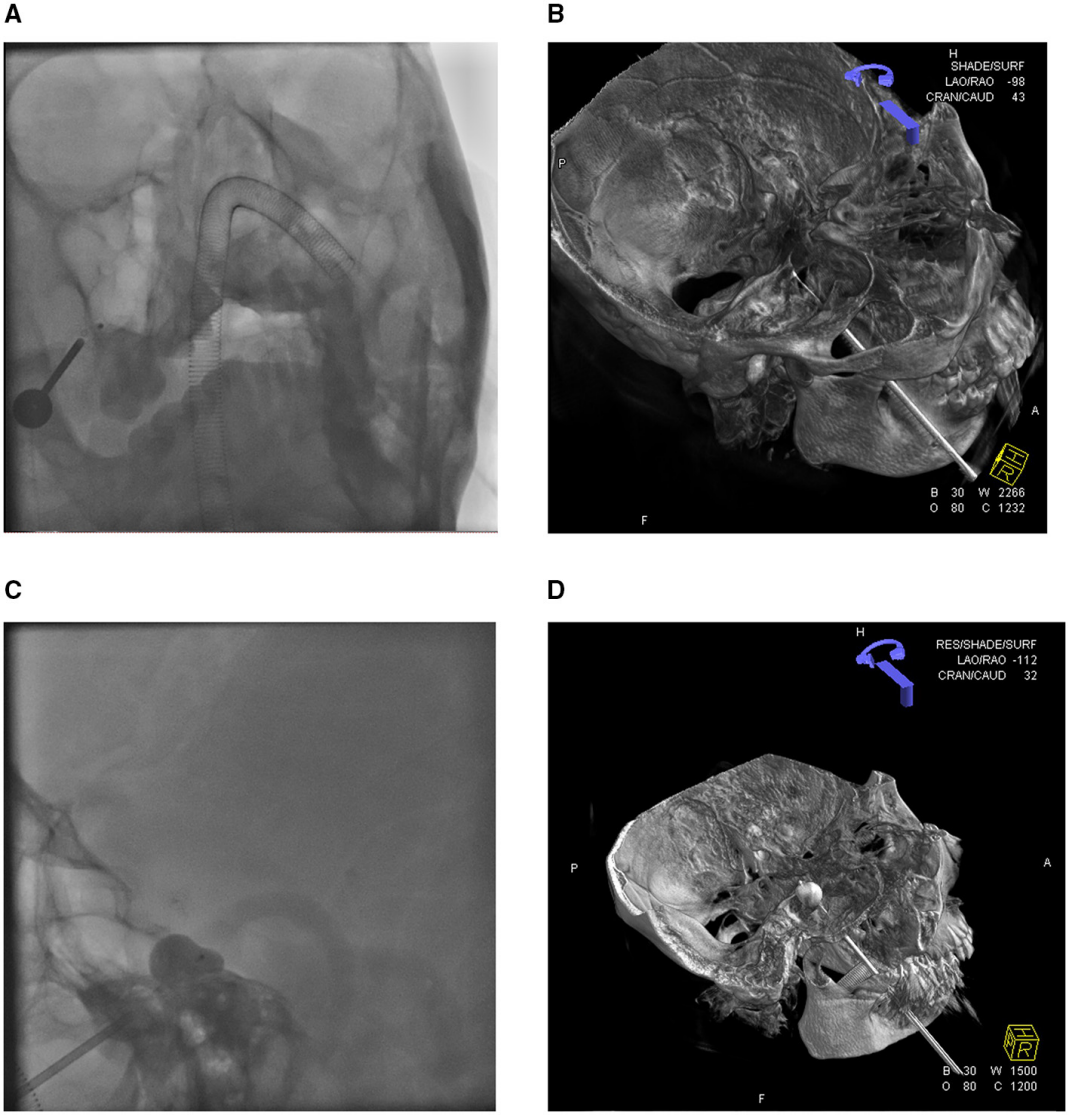
3D-DSA reconstruction displays the position of the balloon catheter. **(A)** Orthography shows the puncture needle and balloon catheter. **(B)** 3D-DSA visualization of balloon catheter placement status. **(C)** The lateral view of the balloon presented the classic “pear shape.” **(D)** 3D-DSA visualization of balloon dilation.

All 40 patients underwent endotracheal intubation under general intravenous anesthesia. The procedures were completed in all cases, with an average surgical duration of approximately 30 min. Bradycardia (heart rate <50 bpm) occurred in 38 patients during the insertion of the puncture needle sheath into the FO. Procedures were suspended when bradycardia occurred and resumed only after recovery to baseline heart rate (>70 bpm). For persistent bradycardia, atropine (0.5 mg) was administered intravenously before continuation. The procedure was immediately terminated and a precordial thump was applied in two patients who developed cardiac arrest. Cardiac rhythm was restored in both patients following electrocardiographic monitoring, with the subsequent resumption of standard surgical protocols, as shown in Figure 1 demonstrating intraoperative 3D DSA navigation.

Post-operatively, 38 patients (95.0%) achieved immediate pain relief, another required 3 days, and another required 2 weeks for complete pain resolution. The mean hospitalization duration was 4.5 ± 1.9 days. All 40 patients experienced facial numbness post-operatively. Complications included perioral herpes in two patients, diplopia in one patient, and localized masseter muscle weakness in one patient, all of which resolved or were well-tolerated within 3 months after treatment. Two patients (5.0%) experienced recurrence during the follow-up (occurring at 4 and 10 months, respectively; Table 2).

**Table 2:** Post-operative data of 40 patients.

**Index**	**Value (*n*)**	**Proportion (%)**
**Time of pain relief**		
The day of operation	38	95.0
3 days after operation	1	2.5
2 weeks after operation	1	2.5
**Complications**		
Facial numbness	40	100
Perioral herpes	2	5.0
Diplopia	1	2.5
Masseter muscle weakness	1	2.5
Recurrence	2	5.0

Pre-operatively, the VAS pain scores of the 40 patients ranged from 7 to 10, with a mean score of 8.6 ± 0.9. Post-operatively, 35 patients (87.5%) achieved complete pain relief (VAS ≤ 1) and three (7.5%) demonstrated significant improvement (occasional pain, VAS >1 to ≤ 3). However, two patients (5.0%) showed no significant relief (no change in the VAS score), resulting in an immediate pain-relief rate of 95.5%. ANOVA revealed significant pain relief at all post-operative time points: immediately and 1 and 3 months post-surgery (*P* < 0.010; Table 3).

**Table 3:** VAS score of patients at each time point (X ± s).

**Time**	**VAS score**
1 day pre-operatively	8.6 ± 0.9
1 day post-operatively	1.2 ± 2.1^*^
1 months post-operatively	0.9 ± 0.7^*^
3 months post-operatively	1.0 ± 0.8^*^

^*^Compared with pre-operative (P <0.010).

VAS, visual analog scale.

## 4 Discussion

The current surgical treatments for trigeminal neuralgia primarily include MVD, PBC, RFT, and stereotactic radiosurgery (7). A prospective study revealed that MVD achieves an 86% complete pain relief rate and a low complication rate, making it an effective intervention. However, this procedure requires advanced surgical expertise and is reserved for cases of definitive vascular compression on imaging studies (8, 9). RFT and stereotactic radiosurgery, which require only local anesthesia, are suitable for patients who are ineligible for general anesthesia. Although RFT achieves a pain relief rate of 82%, it has higher recurrence rates and poorer patient tolerance (10, 11). Stereotactic radiosurgery (Gamma Knife and CyberKnife) offers advantages in alleviating stabbing or post-thermocoagulation pain (1); however, long-term follow-up shows variable early complete remission rates (Gamma Knife: 28.6%−100%; CyberKnife: 40%−72%) and significant recurrence risks (Gamma Knife: 0%−52.2%; CyberKnife: 15.8%−33%) (12).

PBC is used in treating trigeminal neuralgia by injecting a contrast medium to inflate the balloon, physically compressing the trigeminal ganglion. This selectively damages myelinated thick nerve fibers to disrupt pain transmission, while preserving unmyelinated thin nerve fibers that maintain corneal reflex function. Developed for over four decades, PBC has become a highly standardized surgical technique and one of the preferred interventions for trigeminal neuralgia (13). Clinical studies have reported post-operative pain relief rates ranging from 82 to 97.1%. PBC is more suitable for patients who are unable to cooperate intraoperatively owing to anxiety than RFT and stereotactic radiosurgery. Relative to MVD, it benefits patients with contraindications to craniotomy, poor tolerance to open surgery, advanced age, and high quality of life expectations (14). Furthermore, PBC shows favorable efficacy in treating recurrent trigeminal neuralgia following MVD, RFT, or gamma knife radiosurgery, achieving complete pain remission rates of 86% in these refractory cases (15).

PBC for trigeminal neuralgia is predominantly performed under C-arm guidance. Recently, Dyna-CT 3D reconstruction technology, a volumetric imaging modality derived from DSA, has exhibited significant improvements in procedural safety and precision. This technology uses real-time 3D reconstruction algorithms to generate high-resolution spatial visualizations of the FO and its adjacent vascular structures. Dyna-CT enhances puncture accuracy through multi-angle imaging and dynamic navigation in complex anatomical scenarios, including FO stenosis, bony crest prominence, or vascular anomalies. Comparative studies have revealed that traditional C-arm-guided FO puncture exhibits a failure rate of approximately 9.1%; however, Dyna-CT achieves a 100% success rate by enabling pre-operative virtual path simulation and real-time trajectory adjustment (16). The virtual simulation functionality of the system allows operators to predetermine the optimal needle pathways while avoiding critical vasculature, including the internal carotid and maxillary arteries, thereby reducing the risk of intracranial hemorrhage or hematoma formation during balloon placement (17).

In this study, all 40 patients were successfully punctured with short operative times and no severe post-operative complications. During the operation, 38 patients experienced a decrease in heart rate and two experienced cardiac arrest, which is related to the core risk of PBC—trigeminocardiac reflex (TCR). TCR is an acute cardiovascular depression caused by mechanical stimulation of the brainstem vagus nerve pathway during surgery, manifested as a sudden drop in heart rate and sudden increase in blood pressure. However, the incidence of serious complications such as cardiac arrest is low (3), and the risk can be effectively controlled through pre-operative evaluation, anesthesia management, and intraoperative intervention. Pre-operative assessment includes screening high-risk patients for predisposing factors (such as a pre-operative heart rate <60 beats/min and concomitant conduction system disease), avoiding the use of beta blockers and other drugs that worsen TCR, precision anesthesia management including deep anesthesia combined with muscle relaxants, and invasive blood pressure monitoring. Intraoperative intervention involves stopping the operation immediately when TCR occurs, and intravenous injection of atropine if necessary. Through these standardized operational methods, the core risk of PBC can be significantly reduced, providing a relatively high overall security. The early post-operative short-term application of glucocorticoids to reduce nerve edema, supplemented with neurotrophic drugs (methylcobalamin + vitamin B1/B6) to promote nerve myelin repair, combined with physical rehabilitation therapy, if necessary, results in the resolution of post-operative symptoms including facial numbness, perioral herpes, and masseter muscle weakness. A reduction in the post-operative VAS score indicated favorable treatment efficacy and prognosis. This represents a single-center study of PBC under DSA 3D navigation, with more cases reported presently. During operative procedures, the real-time feedback mechanism in Dyna-CT optimizes balloon compression efficacy. Dynamic monitoring of balloon inflation enabled precise adjustments to balloon position and morphology, ensuring the formation of an ideal “pear-shaped” compression zone over the ventral aspect of the trigeminal ganglion—a critical pain conduction region. Non-pear-shaped balloons cause incomplete compression or insufficient selective nerve fiber injury, resulting in post-operative recurrence rates of approximately 31.9%. Conversely, standardized Dyna-CT-guided procedures maintain efficacy rates that exceed 91.7% (5, 16). Quantitative analysis of the balloon pressure and volume has provided individualized treatment parameters to prevent excessive compression-induced masseter weakness or facial dysesthesia (18, 19). Dyna-CT instantly identified intraoperative complications such as balloon rupture (approximately 4.2% incidence), allowing timely adjustments to the compression duration or pressure parameters to reduce the incidence of complications. For complex cases, the multi-modal fusion technology of Dyna-CT has unique advantages. Integrated pre-operative magnetic resonance imaging, computed tomography angiography, and intraoperative data have enabled the comprehensive evaluation of Meckel's cave volume, neurovascular compression severity, and anatomical variations (including trigeminal nerve root dispersion or flattened Meckel's cave), facilitating tailored surgical strategies (16). The 3D reconstruction function simulates angle-specific tissue effects, which specifically benefit older patients and those with comorbidities (such as hypertension and diabetes) (20). Huo et al. (5) and Jain et al. (17) have demonstrated that Dyna-CT-assisted procedures exhibit lower post-operative complication rates than conventional PBC. Dyna-CT increased the intraoperative radiation exposure; nevertheless, the total dose remained controllable with enhanced precision and safety. Multi-modal image fusion accelerates the learning curves of young surgeons and reduces intraoperative adjustments. Future advancements in low-dose scanning technology and artificial intelligence-assisted analysis promise more efficient and personalized Dyna-CT applications in PBC, establishing this technique as an effective trigeminal neuralgia treatment modality.

## 5 Conclusion

Under the guidance of 3D-DSA navigation, PBC for trigeminal neuralgia achieves precise visualization and quantitative assessment through multi-temporal 3D-Dyna-CT dynamic reconstruction. Multimodal imaging fusion enables puncture path-planning of the FO, the real-time dynamic monitoring of puncture pathways and balloon morphology, the visualization of the relative spatial positioning between the balloon and petrous crest, and the elucidation of the spatial relationship between the balloon and Meckel's cave. This method substantially enhances the precision of PBC, improves pear-shaped formation efficiency, reduces intraoperative hemorrhage and other cranial nerve injury complications, and represents a safe, minimally invasive, and technically straightforward approach with favorable clinical applicability.

## Data Availability

The original contributions presented in the study are included in the article/supplementary material, further inquiries can be directed to the corresponding author.
